# Myosteatosis in multiple myeloma: a key determinant of survival beyond sarcopenia

**DOI:** 10.1007/s00256-024-04735-y

**Published:** 2024-06-28

**Authors:** Thierno D. Diallo, Ariane Irma Luise Blessing, Gabriele Ihorst, Mandy Deborah Möller, Pia M. Jungmann, Fabian Bamberg, Georg Herget, Ralph Wäsch, Monika Engelhardt, Jakob Neubauer

**Affiliations:** 1 Department of Diagnostic and Interventional Radiology, University Medical Center Freiburg, Faculty of Medicine, University of Freiburg, Hugstetter Straße 55, 79106 Freiburg, Germany; 2 Clinical Trials Unit, University Medical Center Freiburg, Faculty of Medicine, University of Freiburg, Freiburg, Germany; 3 Department of Medicine I Hematology and Oncology, University Medical Center Freiburg, Faculty of Medicine, University of Freiburg, Freiburg, Germany; 4 Comprehensive Cancer Center Freiburg (CCCF), University Medical Center Freiburg, Faculty of Medicine, University of Freiburg, Freiburg, Germany; 5 Department of Orthopaedics and Trauma Surgery, Faculty of Medicine, Medical Center – University of Freiburg, Freiburg, Germany

**Keywords:** Body Composition, Multiple Myeloma, Myosteatosis, Sarcopenia, CT Imaging

## Abstract

**Objective:**

Fatty infiltration of skeletal muscle (Myosteatosis) is associated with increased frailty, decreased muscle and mobility function, which seems fairly prevalent in multiple myeloma (MM) patients. This study aimed to determine the prognostic value of myosteatosis assessed by CT for progression-free survival (PFS) and overall survival (OS).

**Materials and methods:**

This IRB-approved cohort study included patients with newly diagnosed MM who were treated at a single university hospital and received CT at baseline. Geriatric assessment was performed via International Myeloma Working Group frailty score and Revised Myeloma Comorbidity Index. Myosteatosis was determined through measurement of paravertebral muscle radiodensity. Statistical analyses included uni- and multivariable Cox proportional hazard models and the Kaplan–Meier-method.

**Results:**

A total of 226 newly diagnosed MM patients (median age: 65 years [range: 29–89], 63% males, mean BMI: 25 [14–42]) were analyzed. The prevalence of myosteatosis was 51%. Muscle radiodensity was significantly decreased in individuals with International Staging System stage III vs. I (*p* < 0.001), indicating higher fatty muscle infiltration in patients with advanced disease. Both PFS and OS were significantly decreased in patients with myosteatosis (PFS: median 32.0 months (95% CI 20.5.5–42.2) vs. 66.4 months without myosteatosis (95% CI 42.5-not reached), *p* < .001); OS: median 58.6 (95% CI 51.3—90.2) vs. not reached, *p* < .001).

Myosteatosis remained an independent predictor of OS in multivariable analyses (HR: 1.98; 95%-CI: 1.20–3.27).

**Conclusion:**

Myosteatosis seems fairly prevalent in patients with newly diagnosed MM and associated with impaired overall survival. Prospective clinical trials are required to better understand the role of myosteatosis in MM patients.

**Supplementary Information:**

The online version contains supplementary material available at 10.1007/s00256-024-04735-y.

## Introduction

Multiple myeloma (MM) is a neoplastic plasma cell disease that leads to clonal proliferation of malignant plasma cells in the bone marrow microenvironment [1]. MM is the second most common hematological malignancy, affecting predominantly elderly individuals with a high incidence in patients older than 65 years and the median age at diagnosis of 69 years [2]. Due to demographic changes, its prevalence is likely to increase in the next decades [3]. MM has a high morbidity and mortality as a result of reduced renal function, bone disease and patients' susceptibility to recurrent infections, albeit in general both progression-free (PFS) and overall survival (OS) have impressively increased due to biological insights, earlier diagnosis and better treatment with novel agents, both in younger and older MM cohorts [4, 5]. Nonetheless, therapy management in elderly patients remains challenging, often requiring individualized treatment concepts due to comorbidities, physiological impairment of various organ functions and age-related changes in physical constitution [5]. In order to address these issues and optimize risk stratification, novel frailty risk scores for MM such as the International Myeloma Working Group (IMWG) frailty score, the Revised Myeloma Comorbidity Index (R-MCI) and others have been introduced, complementing the established revised (R-) International Staging System (ISS) [6, 7].

Physical fitness or frailty are known as prognostic factors. In addition, concomitant alterations in body composition also seem to influence the prognosis in several malignant diseases [8]. Body composition analyses using cross sectional imaging and especially Computed tomography (CT) are increasingly applied in oncological research [9]. Single slice measurements of adipose and skeletal muscle (SM) tissue compartments on CT scans at the level of the third lumbar vertebra (L3) have been established as surrogate parameters due to a high correlation with overall body composition [10].

Myosteatosis and sarcopenia are two distinct conditions affecting skeletal muscle, with important differences in their pathophysiology, histopathology, and evaluation on cross sectional imaging [11, 12]. Myosteatosis is characterized by the accumulation of fat within and between muscle fibers, leading to reduced muscle quality and function, while sarcopenia is defined as the (age-) related loss of muscle mass and strength, primarily driven by a decline in the number and size of muscle fibers. Histologically, myosteatosis reveals increased lipid droplets and adipose tissue within the muscle, whereas sarcopenia shows a reduction in muscle fiber cross-sectional area and number [13, 14]. CT can differentiate these conditions, with myosteatosis assessment measuring SM radiodensity, and sarcopenia evaluation relying on muscle cross-sectional area measurements. Several studies have investigated the association between sarcopenia and survival outcomes in patients with multiple myeloma, but the results have been inconsistent and sometimes contradictory [15–17].

Recent literature identifies myosteatosis as a potential prognostic factor for OS in different types of solid cancer [18, 19]. However, data on CT-based myosteatosis and its predictive value are still scarce in regard to hematological cancers, especially MM.

The aim of our study was to evaluate the association of myosteatosis in MM patients with possibly affected PFS and OS.

## Methods

### Patients and clinicopathological data

Approval from the Institutional Review Board was obtained and in keeping with the policies for a retrospective study, informed consent was not required.

We included patients who were treated at our University Comprehensive Cancer Center Freiburg (CCCF) between 01/2000 and 12/2018 and underwent CT imaging at initial diagnosis. Data were retrospectively analyzed as of 12/2018. Patient data were retrieved from our institution's electronic medical records, including sex, age, weight, and BMI. Fluorescence in situ hybridization was performed on bone marrow aspirates of 178 patients (79%, Table 1). This technique targeted the following genetic abnormalities considered most clinically significant, as previously described: del(17p), t(4;14), t(14;16), and t(14;20), which are classified as high-risk or unfavorable. In contrast, t(11;14) and hyperdiploidy are generally considered standard-risk or favorable cytogenetic abnormalities. The presence of del(13q) and c-Myc abnormalities were also evaluated [20]. For each patient, we prospectively assessed frailty via IMWG frailty score and R-MCI. With IMWG frailty scores of 0, 1 or ≥ 2 and R-MCI of 0–3, 4–6 and 7–9 patients were defined as fit, intermediate-fit and frail, respectively. Induction was performed according to European Myeloma Network, German Myeloma Study Group (DSMM) and CCCF standards of bortezomib-induction triplets (i.e. VCD/VRd) plus ASCT, whereas in non-transplant-eligible patients, mostly either bortezomib-based induction (VCD/VD) or IMiD-treatment (Lenalidomide/Dexamethasone; Rd) was performed [5, 20].

**Table 1:** Demographics and characteristics of the study cohort. Data are presented as median [range] for continuous variables and counts and percentages for categorical variables

	Entire population (*n* = 226)	No myosteatosis (*n* = 111) [49%]	Myosteatosis (*n* = 115) [51%]	*p*-value
Median age at initial diagnosis [range]	65 [29–89]	62 [29–84]	69 [42–89]	** < *.001***
Sex				
Male (%)	142 (63%)	38 (27%)	104 (73%)	** < *.001***
Female (%)	84 (27%)	73 (87%)	11 (13%)	
BMI [range]	24.7 [14.1–42.5]	24.4 [14.1–40.1]	24.9 [15.2–42.5]	*0.17*
R-MCI				** < *.001***
0–3 = fit	48 (21%)	35 (73%)	13 (27%)	
4–6 = intermediate-fit	128 (57%)	59 (46%)	69 (54%)	
7–9 = frail	50 (22%)	17 (34%)	33 (66%)	
IMWG frailty score				** < *.001***
0 = fit	74 (33%)	47 (64%)	27 (36%)	
1 = intermediate-fit	66 (29%)	33 (50%)	33 (50%)	
≥ 2 = frail	86 (38%)	31 (36%)	55 (64%)	
Cytogenetic risk				*0.53*
low	94 (42%)	47 (50%)	47 (50%)	
high	84 (37%)	43 (51%)	41 (49%)	
unknown	48 (21%)	21 (44%)	27 (56%)	
IgG type	123 (55%)	68 (55%)	55 (45%)	***0.04***
ISS				***0.01***
I	82 (36%)	52 (63%)	30 (37%)	
II	61 (27%)	27 (44%)	34 (56%)	
III	83 (37%)	32 (39%)	51 (61%)	
ASCT	131 (58%)	77 (59%)	54 (41%)	** < *.001***

*Abbreviations*: *BMI* Body-Mass-Index; *R-MCI* Revised Myeloma Comorbidity Index; *IMWG* International Myeloma Working Group; *ASCT* autologous stem cell transplantation; *ISS* International Staging System

### Imaging analysis

**Fig. 1:**
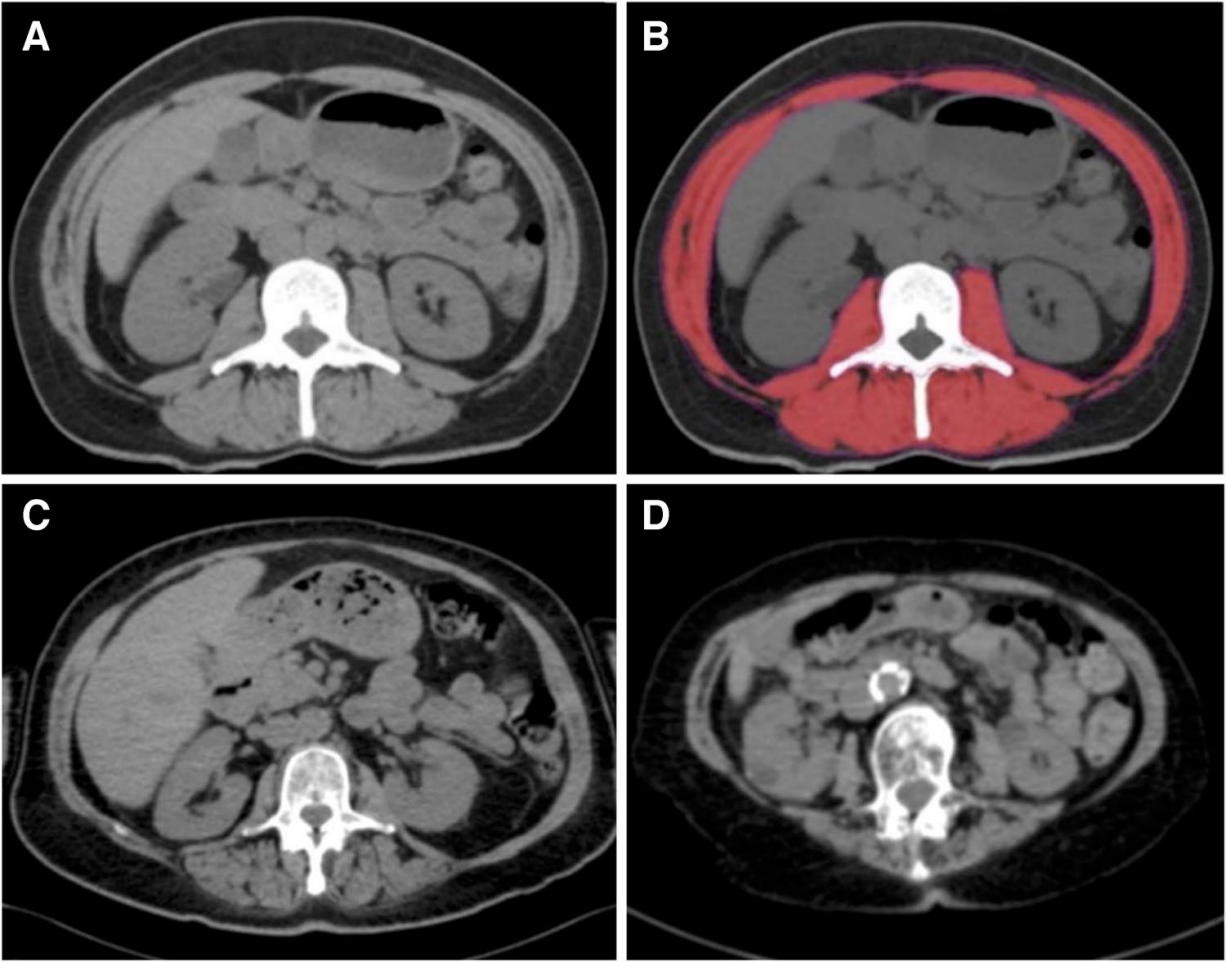
Cross-sectional CT images at the level of the third lumbar vertebra in patients with myosteatosis and sarcopenia. **A** Axial CT image at the level of the third lumbar vertebra identified for analysis. **B** Red area indicating segmentation of skeletal muscle. **C** Myosteatosis in a 64-year-old woman (MA: 19 HU). **D** Sarcopenia in a 72-year-old woman (SMI: 23 cm^2^/m^2^). Abbreviations: CT: Computed Tomography; HU: Hounsfield Units; MA: muscle attenuation; SMI: skeletal muscle index

Abdominal non-contrast CT scans of routinely performed baseline staging examinations were obtained from our Picture Archiving and Communication System. A single CT image slice at L3 level was identified for analysis as previously described (Fig. 1) [10].

Images were primarily analyzed by a single trained investigator in a random order, using Aquarius iNtuition (Aquarius iNtuition viewer, version 4.4, TeraRecon, San Mateo, CA, USA).

Hereby, SM was automatically segmented using Hounsfield unit (HU) thresholds (-29 – 150 HU). The mean HU values of all SM tissue within the L3 cross-section were recorded to evaluate myosteatosis.

**Fig. 2:**
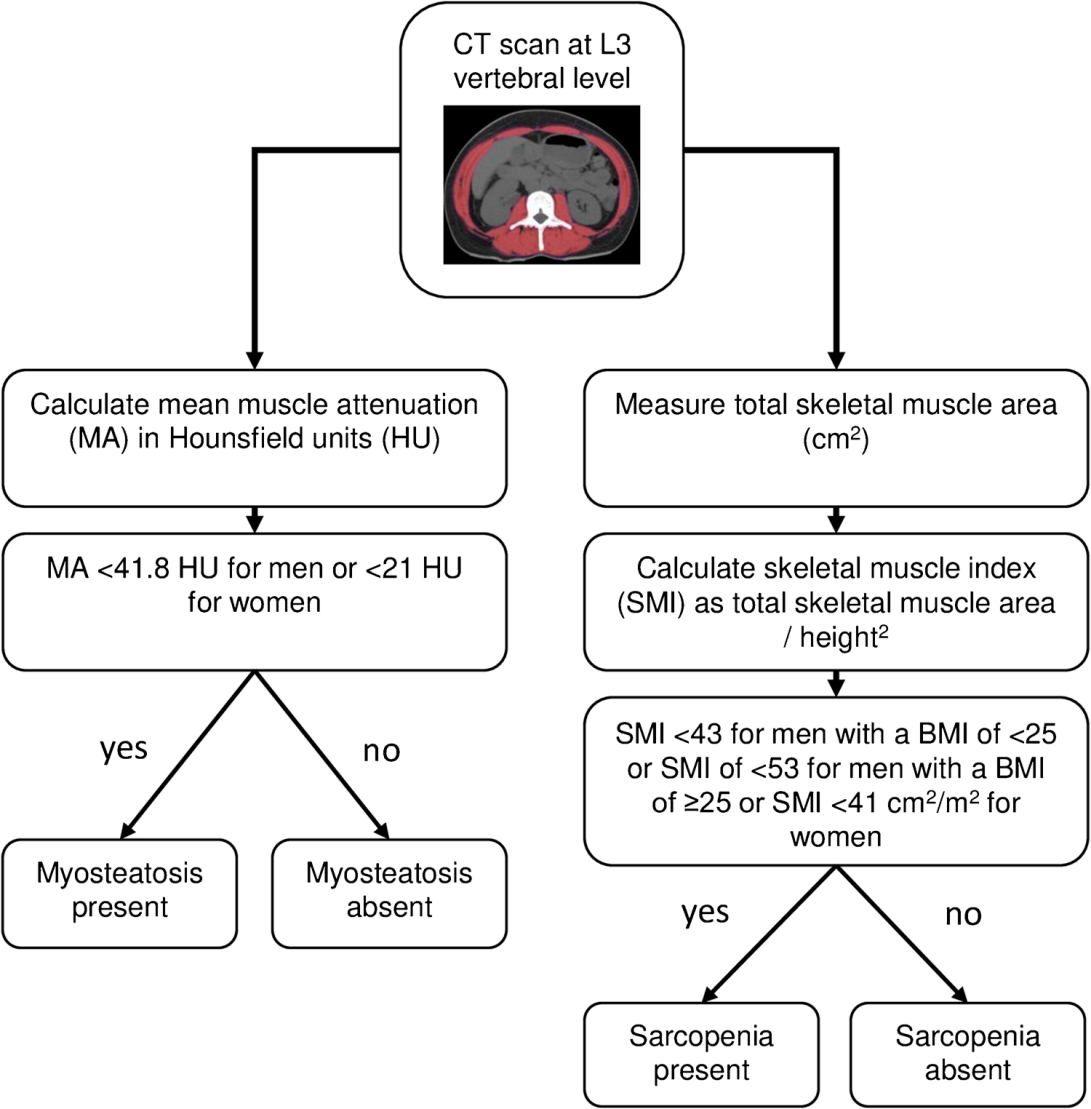
Flow diagram: skeletal muscle measurements. Abbreviations: MA: muscle attenuation; HU: Hounsfield Units; SMI: skeletal muscle index; BMI: Body-Mass-Index

Further, the SM area was normalized to body height in cm^2^/m^2^ to calculate the skeletal muscle index (SMI) and determine the presence or absence of sarcopenia. Sarcopenia was defined as SMI of < 43 for men with a BMI of < 25, SMI of < 53 for men with a BMI of ≥ 25, and SMI of < 41 for women, according to the widely used definition by Martin et al. (Fig. 2) [21].

All segmentation results were then reviewed in consensus by the trained investigator and a board-approved radiologist with 10 years of experience in oncologic imaging and corrected if necessary. Patients were excluded from the study if segmentation was not possible.

Both readers (TD, JN) were fully blinded to patients' clinicopathological data at the time of evaluation.

### Statistical analysis

Statistical analyses were performed using SAS version 9.2 (SAS Institute Inc.) and R, Version 4.0.5 (A Language and Environment for Statistical Computing, R Foundation for Statistical Computing, http://www.R-project.org). Continuous data were reported as median values [ranges] and categorical data were presented as proportions. Mann–Whitney-U and χ2 tests were used to test for differences in continuous and categorical variables, respectively. Comparison of mean SM radiodensity with regard to ISS stages and frailty scores was conducted using univariable analysis of variance (ANOVA). The optimal sex stratified SM radiodensity cut-off values for determining myosteatosis were calculated using the "survminer" (https://cran.r-project.org/web/packages/survminer/index.html) and "survival" (https://cran.r-project.org/web/packages/survival/index.html) packages in R (version 4.0.5). The "surv_cutpoint" function from the "survminer" package was employed to determine the optimal cut-off point for SM radiodensity based on the survival data, maximizing the difference in survival between the resulting groups. The identified optimal SM radiodensity cut-off value was then used to categorize patients as having either normal muscle or myosteatosis, allowing for the comparison of survival outcomes between these two groups.

PFS and OS were calculated as time from diagnosis to death from any cause and first observation of relapse or death, and were analyzed using the Kaplan–Meier method, with differences between groups evaluated using the log-rank test. Univariable and multivariable analyses for PFS and OS were conducted using the Cox proportional hazards model, hazard ratios (HRs) and corresponding 95% confidence intervals (CIs).

Univariable analyses were performed to assess the prognostic significance of CT body composition parameters (myosteatosis and sarcopenia) and other potential prognostic factors, including age (continuous), sex, BMI (continuous), cytogenetic risk (high risk), autologous stem cell transplantation (ASCT) status, multiple myeloma type (IgG), International Staging System (ISS) stage (II vs. III), International Myeloma Working Group (IMWG) frailty score, and Revised Myeloma Comorbidity Index (R-MCI) score (fit vs. intermediate vs. frail). Variables for the multivariable Cox regression models were selected based on their clinical and prognostic relevance, as determined by previous literature, while respecting the degrees of freedom to avoid overfitting.

Multivariable models were constructed to assess the prognostic value of body composition parameters and the robustness of results, while adjusting for the selected variables, including risk stratification systems. Models 1 (PFS) and 2 (OS) included myosteatosis, sarcopenia, age, ASCT status, cytogenetic risk, and multiple myeloma type (IgG).

Models 3 (PFS) and 4 (OS) included myosteatosis, sarcopenia, ISS stage, and the IMWG frailty score or alternatively R-MCI score.

All analyses were performed using two-tailed testing and differences were considered significant at p ≤ 0.05.

## Results

### Patient characteristics

**Fig. 3:**
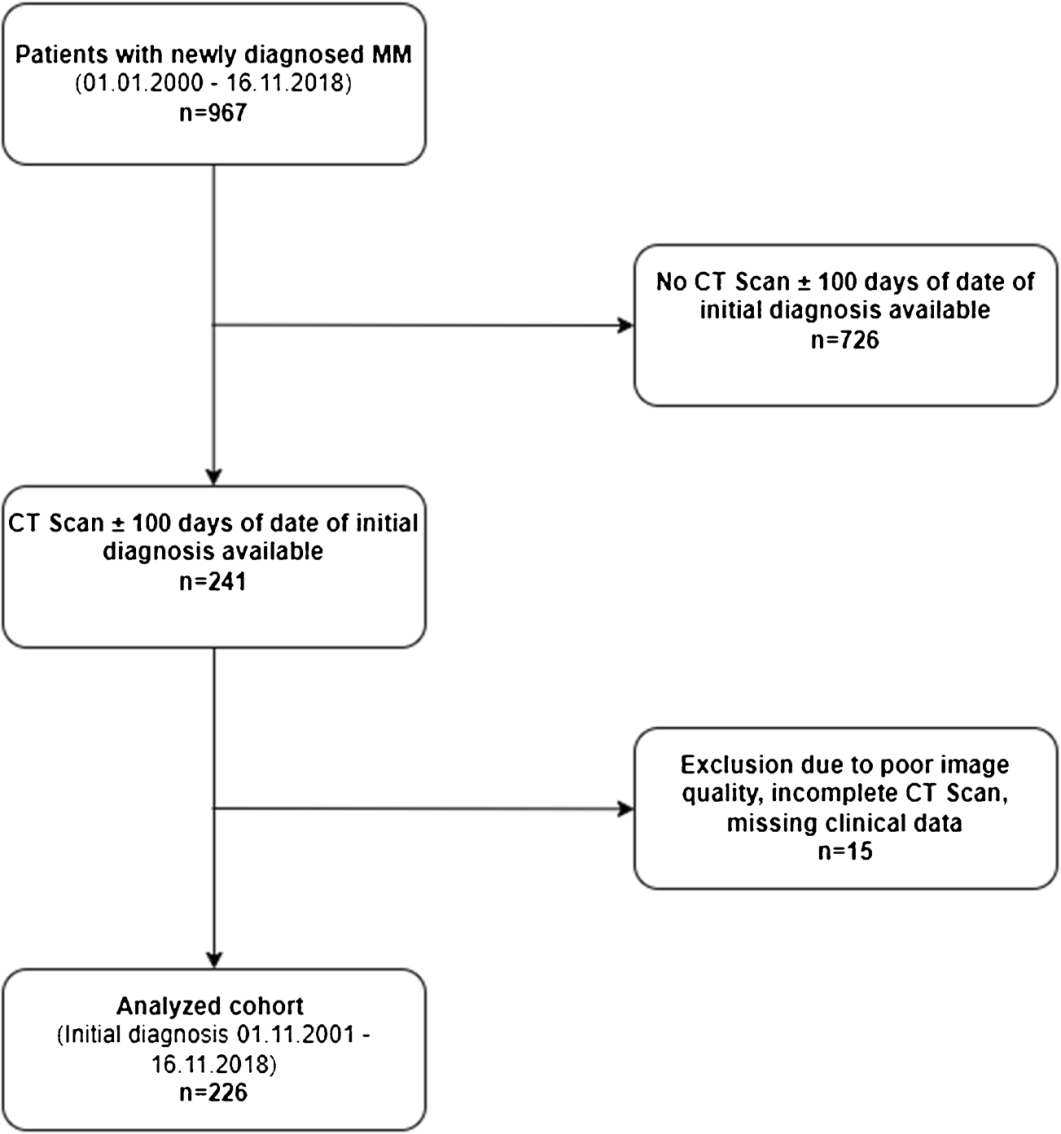
Flow diagram: patient selection. Abbreviations: MM: multiple myeloma; CT: Computed Tomography

A total of 226 patients were analyzed in this study (Fig. 3), with a median follow-up of 54 months. Patients´ baseline and clinical characteristics are summarized in Table 1. The median age was 65 years [range: 29–89]. The majority of the patients were male (63%). The median BMI was 24.7 kg/m^2^ [range: 14.1–42.5]. According to the ISS staging system, 82 (36%), 61 (27%) and 83 (37%) patients had ISS stage I, II and III, respectively. Expectedly, the most prevalent paraprotein MM-subtype was IgG MM in 123 patients (55%). Ninety-four (42%) patients had more favorable cytogenetics, whereas 84 (37%) had unfavorable cytogenetics. For the remaining 48 patients (21%) no data on cytogenetics were available (because referral patients from outside private-practices for second opinion and therapy recommendations had either not performed or stored the results in our electronic data system).

### Prevalence of myosteatosis

The optimal stratification analysis yielded the following cut-off values for myosteatosis: 41.8 HU for male, and 21.0 HU for female individuals, respectively. Thus, individuals below the determined cut-off values were classified into the myosteatosis group.

The overall prevalence of myosteatosis was 51% (*n* = 115/226). Differences of patients with and without myosteatosis are depicted in Table 1. Individuals with myosteatosis were older than non-myosteatotic individuals (69 years [range: 42–89] vs. 62 years [29–84], *p* < 0.001).

The proportion of men was higher in the group with myosteatosis (*p* < 0.001). BMI showed no difference between individuals with myosteatosis compared to non-myosteatotic patients (24.9 [15.2–42.5]. vs. 24.4 [14.1–40.1]; *p* = 0.17). As expected, the proportion of patients receiving ASCT was significantly lower in the myosteatoic group (41% vs. 59% in those without myosteatosis, *p* < 0.001). In line, patients with myosteatosis were more often intermediate-fit or frail, according to both IMWG frailty and R-MCI scores (both *p* < 0.001, respectively, Table 1).

**Fig. 4:**
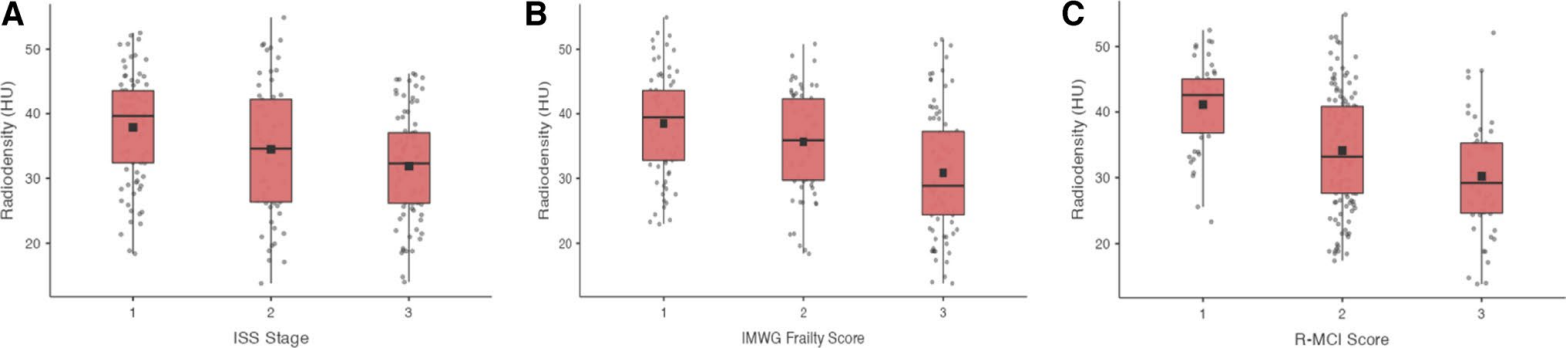
Mean skeletal muscle HU in relation to risk stratification systems. **A** Comparison of mean SM HU in relation to ISS stages. **B** Comparison of mean SM HU in relation to the IMWG frailty score (1: fit, 2: intermediate-fit, 3: frail). **C** Comparison of mean SM HU in relation to the R-MCI score (1: fit, 2: intermediate-fit, 3: frail). Abbreviations: SM: skeletal muscle; HU: Hounsfield Units; ISS: International Staging System; IMWG: International Myeloma Working Group; R-MCI: Revised Myeloma Comorbidity Index

Overall, mean SM HU were significantly lower for ISS stage III compared to ISS stage I (Fig. 4: 31.9 vs. 37.9, *p* < 0.001). Analogously, SM HU showed a decrease from fit to frail patients in the IMWG frailty score (41.1 vs. 30.2), and R-MCI (38.5 vs. 30.9), respectively (both *p* < 0.001).

### Myosteatosis is associated with impaired PFS and OS

**Fig. 5:**
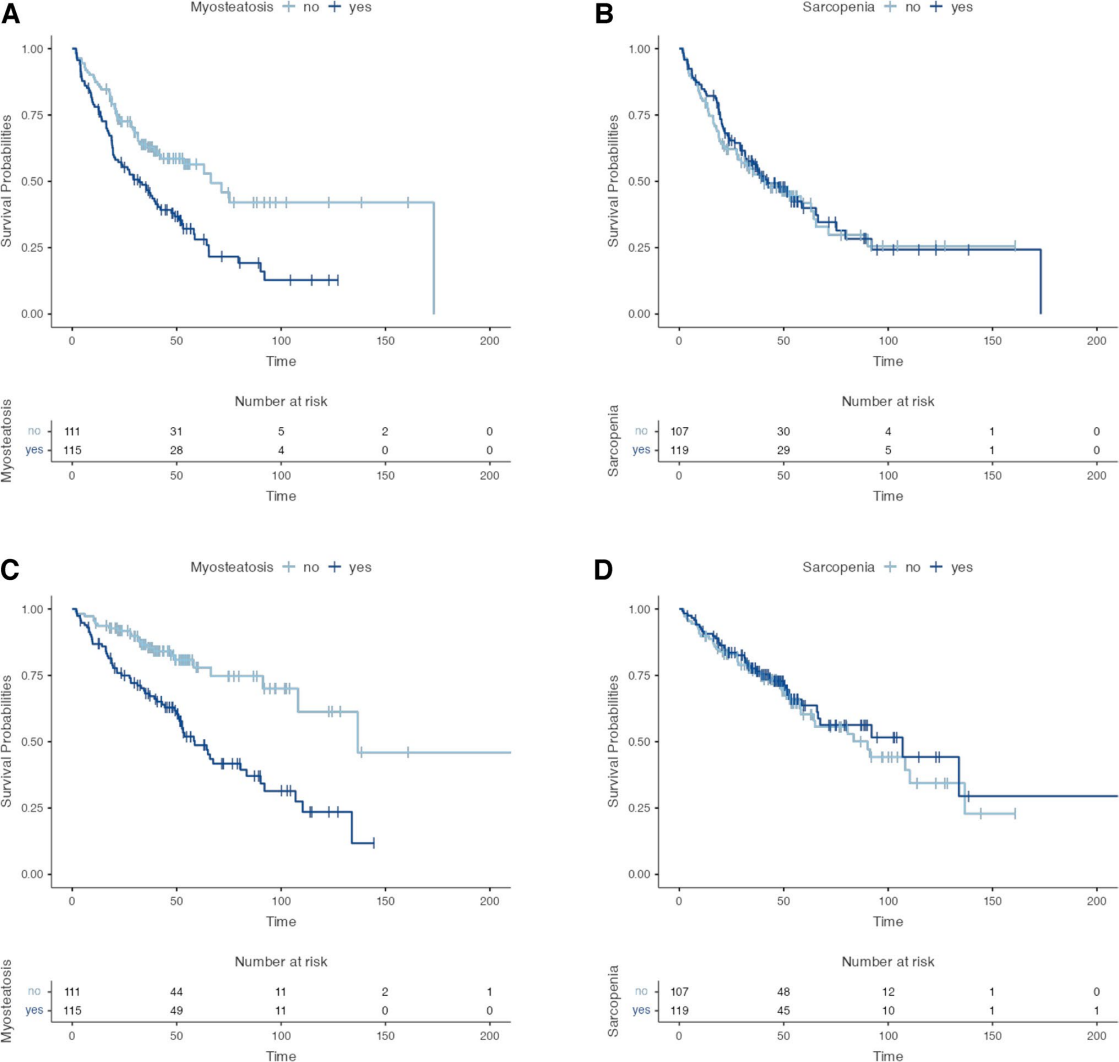
Kaplan–Meier plots. **A** PFS myosteatoic vs. non-myosteatoic. **B** PFS sarcopenic vs. non-sarcopenic. **C** OS myosteatoic vs. non-myosteatoic. **D** OS sarcopenic vs. non-sarcopenic. Abbreviations: PFS: progression-free survival; OS: overall survival

Kaplan–Meier plots for OS and PFS are depicted in Fig. 5A-D and supplementary Fig. 1A + B. Median PFS was 41.0 months (95% CI [35.4–58.6]) and median OS was 91.3 months (95% CI [66.4 -137.0]) for the entire cohort (Supplementary Fig. 1A + B). PFS was impaired in patients with myosteatosis, with a median of 32.0 months (95% CI [20.5.5–42.2]) vs. 66.4 months without myosteatosis (95% CI [42.5-not reached]), respectively (*p* < 0.001; Fig. 5A). Likewise, OS was decreased in patients with myosteatosis (58.6 months (95% CI [51.3—90.2]) vs. 136.6 months (95% CI [108.1-not reached]); *p* < 0.001; Fig. 5C. No evidence of a difference for PFS (42.2 months (95% CI [32.3–66.4]) vs. 41.0 months (95% CI [28.3–65.4]), *p* = 0.69) and OS (106.9 months (95% CI [47.6–166.2]) vs. 90.2 months (95% CI [59.3 -121.2]), *p* = 0.58) was noted for patients with or without sarcopenia, respectively Fig. 5B + D).

### Multivariable analyses show myosteatosis to be an independent predictor of OS

Cox regression for PFS and OS was performed to adjust the effect of myosteatosis for relevant risk factors (Tables 2, 3, 4, and 5).

**Table 2:** Uni- and multivariable Cox regression models for progression-free survival (PFS)

Analysis		Univariable		Multivariable	
Covariate		HR (95% CI)	*P*-value	HR (95% CI)	*P*-value
Age		1.04 (1.02 – 1.06)	** < *.001***	1.01 (0.99 – 1.04)	*0.23*
Sex	*Male*	1.34 (0.92 – 1.93)	*0.13*		
BMI		0.98 (0.94 –1.02)	*0.29*		
Cytogenetic risk	*high*	1.88 (1.25 – 2.85)	***0.003***	1.69 (1.11 – 2.56)	***0.02***
ASCT	*yes*	0.45 (0.32 – 0.65)	** < *.001***	0.66 (0.41 – 1.07)	*0.09*
Immunoglobuline	*IgG type*	0.90 (0.63 – 1.28)	*0.57*	0.97 (0.67 – 1.41)	*0.89*
Myosteatosis	*yes*	1.95 (1.35 – 2.80)	** < *.001***	1.37 (0.86 – 2.18)	*0.18*
Sarcopenia	*yes*	0.93 (0.66 – 1.33)	*0.71*	1.12 (0.77 – 1.61)	*0.56*

*Abbreviations*: *BMI* Body-Mass-Index; *ASCT*: autologous stem cell transplantation; *ISS*: International Staging System

**Table 3:** Uni- and multivariable Cox regression models for overall survival (OS)

Analysis		Univariable		Multivariable	
Covariate		HR (95% CI)	*P*-value	HR (95% CI)	*P*-value
Age		1.06 (1.03 – 1.08)	** < *.001***	1.02 (1.00 – 1.05)	*0.09*
Sex	*Male*	1.15 (0.73 – 1.79)	*0.55*		
BMI		1.00 (0.95 –1.05)	*0.94*		
Cytogenetic risk	*high*	1.97 (1.17 – 3.30)	***0.01***	1.65 (0.98 – 2.80)	*0.06*
ASCT	*yes*	0.28 (0.18 – 0.44)	** < *.001***	0.47 (0.27 – 0.83)	***0.01***
Immunoglobuline	*IgG type*	0.64 (0.42 – 0.99)	***0.04***	0.74 (0.47 – 1.16)	*0.19*
Myosteatosis	*yes*	2.86 (1.77 – 4.64)	** < *.001***	1.98 (1.20 – 3.27)	***0.01***
Sarcopenia	*yes*	0.89 (0.58 – 1.36)	*0.59*	1.11 (0.71 – 1.72)	*0.65*

*Abbreviations*: *BMI*: Body-Mass-Index; *ASCT*: autologous stem cell transplantation; *ISS*: International Staging System

**Table 4:** Uni- and multivariable Cox regression models for progression-free survival (PFS) including risk stratification systems

Analysis		Univariable		Multivariable	
Covariate		HR (95% CI)	*P*-value	HR (95% CI)	*P*-value
Myosteatosis	*yes*	1.93 (1.35 – 2.77)	** < *.001***	1.47 (0.98 – 2.08)	*0.07*
Sarcopenia	*yes*	0.93 (0.66 –1.32)	*0.69*	0.72 (0.50 –1.04)	*0.08*
IMWG Frailty Score	*1 point*	2.18 (1.29 –3.67)	***0.003***	1.88 (1.10 – 3.20)	***0.02***
	≥ *2 points*	3.53 (2.21 – 5.65)	** < *.001***	2.38 (1.44 – 3.93)	***0.001***
ISS	*Stage II*	2.41 (1.46 – 3.98)	***0.001***	2.07 (1.24 – 3.47)	***0.01***
	*Stage III*	3.78 (2.38 – 6.02)	** < *.001***	2.90 (1.75 – 4.80)	** < *.001***
Myosteatosis	*yes*	1.93 (1.35 – 2.77)	** < *.001***	1.37 (0.94 – 2.00)	*0.1*
Sarcopenia	*yes*	0.93 (0.66 –1.32)	*0.69*	0.70 (0.48 –1.01)	*0.06*
R-MCI	*4 – 6 points*	3.04 (1.68 –5.50)	** < *.001***	2.33 (1.26 – 4.32)	***0.01***
	*7 – 9 points*	6.06 (3. 32 –11.34)	** < *.001***	3.88 (1.99 – 7.58)	** < *.001***
ISS	*Stage II*	2.41 (1.46 – 3.98)	***0.001***	1.68 (0.88 – 3.23)	*0.12*
	*Stage III*	3.78 (2.38 – 6.02)	** < *.001***	2.27 (1.21 – 4.27)	***0.01***

*Abbreviations*: *BMI*: Body-Mass-Index; *IMWG*: International Myeloma Working Group; *ISS*: International Staging System; *R-MCI*: Revised Myeloma Comorbidity Index

**Table 5:** Uni- and multivariable Cox regression models for overall survival (OS) including risk stratification systems

Analysis		Univariable		Multivariable	
Covariate		HR (95% CI)	*P*-value	HR (95% CI)	*P*-value
Myosteatosis	*yes*	2.87 (1.77 – 4.65)	** < *.001***	2.21 (1.35 – 3.61)	***0.01***
Sarcopenia	*yes*	0.89 (0.58 –1.36)	*0.58*	0.78 (0.50 –1.22)	*0.28*
IMWG Frailty Score	*1 point*	2.49 (1.23 –5.03)	***0.01***	1.98 (0.96 – 4.07)	*0.06*
	≥ *2 points*	4.57 (2.43 – 8.61)	** < *.001***	2.89 (1.47 – 5.67)	***0.002***
ISS	*Stage II*	2.34 (1.25 – 4.39)	***0.01***	1.79 (0.93 – 3.45)	*0.08*
	*Stage III*	3.64 (2.04 – 6.50)	** < *.001***	2.30 (1.21 – 4.38)	***0.01***
Myosteatosis	*yes*	2.87 (1.77 – 4.65)	** < *.001***	2.04 (1.24 – 3.35)	***0.01***
Sarcopenia	*yes*	0.89 (0.58 –1.36)	*0.58*	0.72 (0.46 –1.13)	*0.16*
R-MCI	*4 – 6 points*	4.52 (1.79 –11.38)	***0.001***	3.04 (1.17 – 7.89)	***0.02***
	*7 – 9 points*	8.68 (3.39 –22.25)	** < *.001***	4.93 (1.82 – 13.33)	***0.002***
ISS	*Stage II*	2.34 (1.25 – 4.39)	***0.01***	1.68 (0.88 – 3.23)	*0.12*
	*Stage III*	3.64 (2.04 – 6.50)	** < *.001***	2.27 (1.21 – 4.27)	***0.01***

*Abbreviations*: *BMI*: Body-Mass-Index; *IMWG*: International Myeloma Working Group; *ISS*: International Staging System; *R-MCI*: Revised Myeloma Comorbidity Index

Myosteatosis showed no independent association with PFS via multivariable testing (HR 1.37, 95% CI [0.86–2.18]; *p* = 0.18). High cytogenetic risk was a predictor of decreased PFS (HR 1.69, 95% CI [1.11–2.56; *p* = 0.02).

For OS, however, myosteatosis was associated with a significant risk, independent of age, high cytogenetic risk, ASCT, MM protein type, and sarcopenia (HR 1.98, 95% CI [1.20–3.27]; *p* = 0.01) (Table 3). Myosteatosis confirmed its independent negative association with OS in the multivariable cox regression models including the MM risk stratification systems (HR 2.04, 95% CI [1.24–3.35]; *p* = 0.01), and (HR 2.21, 95% CI [1.35–3.61]; *p* = 0.01), respectively) (Table 5).

## Discussion

The present study examined the potential impact of muscle depletion, assessed by CT, on survival in patients with MM. The presence of myosteatosis at baseline was significantly associated with impaired OS, independently of relevant risk factors, as well as advanced ISS stages II/III or increased frailty scores for intermediate-fit or frail patients. Furthermore, patients with frailty and/or more advanced MM disease showed lower SM radiodensity, implicating higher amounts of muscular lipid accumulation in these subgroups. No association of sarcopenia with survival was observed in our patient cohort.

To the best of our knowledge only one other study by da Cunha et al. investigated the association of myosteatosis with survival outcome in MM patients.[22]. The study found no association between myosteatosis, according to a previously published cut-off by Martin et al., and survival [21, 22]. Intriguingly, low SM radiodensity, recorded as continuous HU values, showed a trend towards reduced OS. In contrast to our study in Central European MM patients, the study by da Cunha et al. was conducted in a relatively small Latin American cohort (*n* = 84) [22]. Since body composition differs between ethnic groups and possibly different malignant diseases, the cut-off values for myosteatosis by Martin et al., established from a large North American cohort with a substantial number of gastrointestinal malignancies, may not be applicable in all scenarios [23–25]. Taking this into account, we defined cohort specific cut-off values for myosteatosis, stratified by sex, as previously recommended [26].

The impact of body composition on the prognosis of malignant diseases has been a topic of extensive research. In particular, sarcopenia has been widely investigated and associated with adverse outcomes in patients with cancer [27–29]. However, with regard to MM, several studies have found no association between CT-based sarcopenia and MM outcome, which is in line with our observations.[15, 30–32].

Interestingly, emerging evidence suggests that CT-based myosteatosis may be a superior prognostic marker for survival in patients with cancer compared to CT-based sarcopenia, also corroborating our findings [33–36]. Moreover, myosteatosis has also been linked to various other adverse health outcomes, including increased risk of falls, fractures, functional impairment, reduced treatment tolerance and increased toxicity [37–39]. These associations are thought to be mediated by systemic inflammation, metabolic dysregulation, and reduced physical function, all of which can contribute to a generalized decline in patients' overall health status, the latter being a common observation also in individuals with sarcopenia [25, 40]. Notably, despite their pathophysiological differences, sarcopenia and myosteatosis share several common risk factors, such as age, physical inactivity, malnutrition, and chronic inflammation, which may contribute to the development and progression of both conditions [40, 41]. However, myosteatosis represents an early change of SM composition and is considered as a more sensitive marker of muscle degradation, preceding muscle mass and overall body mass loss, while being associated with a significantly decreased muscle quality. [42, 43] This symptom constitutes a part of the frailty syndrome [44]. Compared to CT-based sarcopenia, myosteatosis seems to have a stronger association with frailty, suggesting that qualitative, rather than quantitative changes of SM may have a bigger impact on the development of frailty in elderly individuals with cancer. In line, Williams et al. were able to show that the measurement of SM wasting correlates better with muscle function than the pure measurement of SM area [45]. In contrast to the imaging-based definition of sarcopenia, the current clinical definition of the European Working Group on Sarcopenia in Older People includes impaired muscle function in addition to pure muscle mass loss, emphasizing qualitative muscle changes as important part of the disease [41].

Geriatric assessment and the determination of the patients’ frailty status are part of the current clinical routine and therapy management of patients with MM at our and other cancer centers, allowing a better risk stratification and supporting therapy decisions [46]. In our study population, higher grades in the IMWG-frailty and R-MCI scores were associated with decreased PFS and OS, confirming prior data. Expectedly, patients with myosteatosis were less often receiving stem cell transplants (ASCT) than patients without myosteatosis. Since the decision to ASCT depends more on the fitness of patients than on rigid age values, these results underline the suspected role of myosteatosis in relation to poor physical fitness.

The discrepancy between the associations of myosteatosis with OS and PFS further suggests myosteatosis to be may marker of overall health status and frailty, rather than a direct indicator of disease progression in multiple myeloma, as it may impact patients' ability to tolerate treatment and cope with the overall burden of the disease, leading to worse OS, even if the disease itself is not progressing more rapidly.

Regular physical activity has been suggested to be associated with a better treatment tolerance and less comorbidities in patients with MM undergoing ASCT [47]. Furthermore, regular exercise seems to have a positive effect on myosteatosis, leading to an improvement of SM quality [48]. Treatment in MM is shifting towards more personalized concepts, where risk stratification plays a crucial role. A valuable imaging biomarker may therefore help to define patients at risk and complement the already established risk factors. In 2014, the IMWG included CT as standard imaging modality in the diagnostic algorithm for MM [49]. Since CT represents the current gold standard for body composition analysis, assessment of myosteatosis could routinely be performed on baseline CT scans providing a cost effective, readily available added information on the patients’ constitution, potentially supporting therapy decisions and leading to the initiation of preventive measures, such as regular physical exercise.

Of note, although sarcopenia and myosteatosis may occur simultaneously, the two phenomena are not directly linked or necessarily associated with each other [50]. While the loss of muscle mass may be noticeable to a trained radiologist, the loss of muscle density is a less conspicuous imaging feature. Therefore, a quantitative evaluation will always be the basis for a reliable assessment of this variable.

Our analysis can be criticized for its limited number of MM patients, hence the predictive value of myosteatosis raised in this analysis should be verified in even larger collectives than ours and in the context of prospective studies. Second, the median follow-up duration of 54 months may seem short compared to the median overall survival of 91 months. However, this follow-up period allowed to capture early and intermediate outcomes crucial for understanding myosteatosis' impact on survival. The statistically significant association between myosteatosis and OS suggests that the follow-up duration was sufficient to detect meaningful differences. Nonetheless, longer follow-up data would provide additional insights, and future studies with extended follow-up periods could help validate and expand upon our findings.

Further, our cohort included tertiary and referral patients from a Comprehensive Cancer Center with a substantial proportion of patients with unfavorable cytogenetics (37%). On top, evaluation of SM fat was based on CT attenuation and our results were not compared to histopathology, a direct method for visualization and quantification of fat tissue. However, muscle radiation attenuation is considered linearly dependent on muscle fat content. Finally, imaging evaluation was performed retrospectively since body composition analysis is not part of the clinical routine; therefore CT-readers were blinded to the clinicopathological data.

In conclusion, our results suggest a negative association of myosteatosis with OS in patients with MM. SM depletion through accumulation of adipose tissue might reflect patients’ poor physical constitution and frailty better than the pure loss of muscle mass, expressed by CT-based sarcopenia. Thus, assessment of muscle density via CT imaging may have the potential to add valuable prognostic information and establish myosteatosis as an objective imaging biomarker in patients with MM. Subsequent prospective studies will have to confirm our findings.

## Supplementary Information

Below is the link to the electronic supplementary material.

ESM 1 (PNG 162 kb)

High Resolution Image (TIF 630 kb)
