# Supplementary figures and images for: Myosteatosis in multiple myeloma: a key determinant of survival beyond sarcopenia
Source: Skeletal Radiol. 2024 Jun 28;54(2):275–85. doi: 10.1007/s00256-024-04735-y (PMC11652573; doi:10.1007/s00256-024-04735-y)

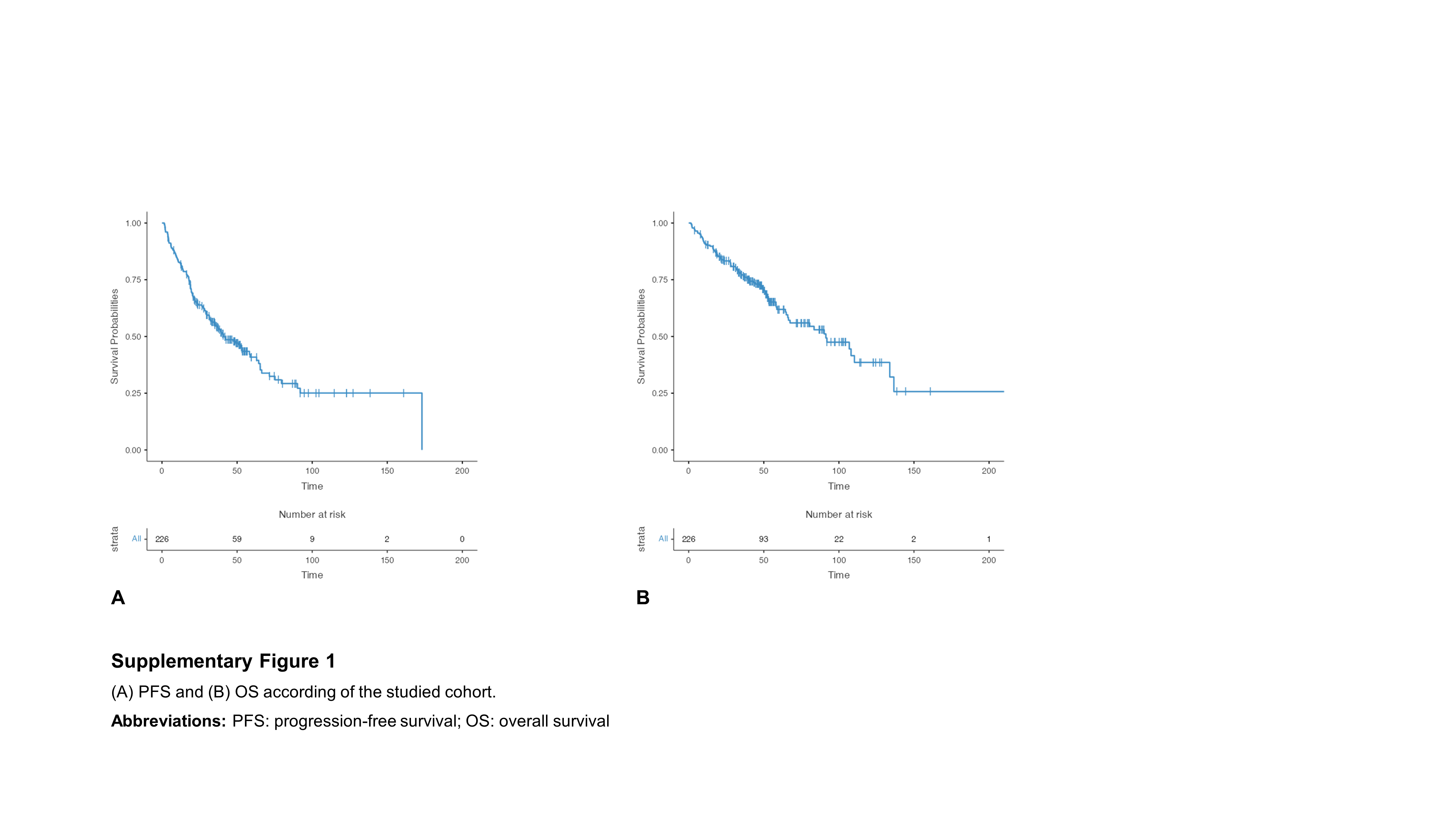

Supplement: Supplementary file 1 — ESM 1 (PNG 162 kb) [file 256_2024_4735_Fig6_ESM.png]

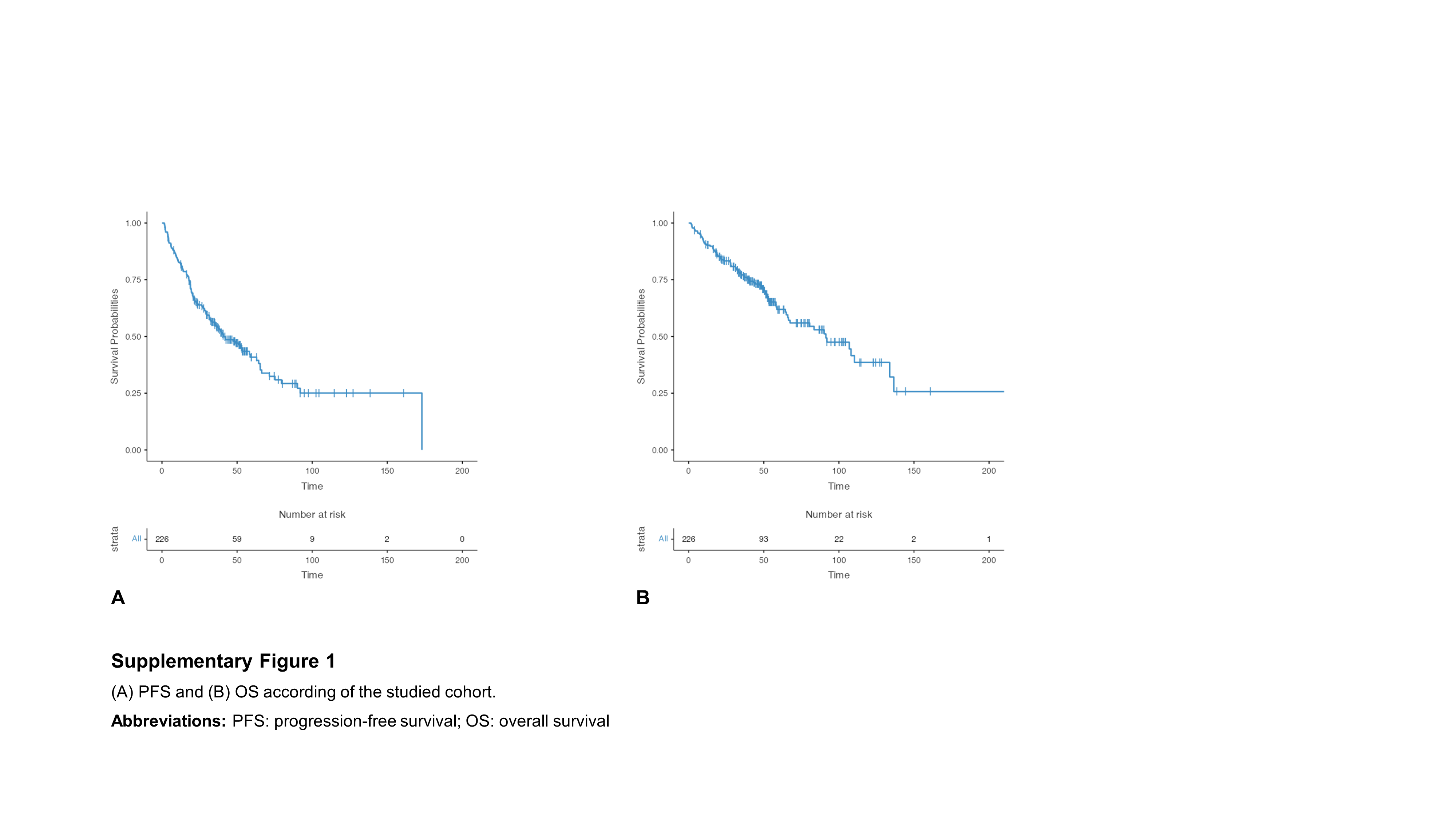

Supplement: Supplementary file 2 — High Resolution Image (TIF 630 kb) [file 256_2024_4735_MOESM1_ESM.tif]
